# Draft Genome Sequence of the Plant Pathogen *Dickeya zeae* DZ2Q, Isolated from Rice in Italy

**DOI:** 10.1128/genomeA.00905-13

**Published:** 2013-11-07

**Authors:** Iris Bertani, Daniel Passos da Silva, Pamela Abbruscato, Pietro Piffanelli, Vittorio Venturi

**Affiliations:** Bacteriology Group, International Centre for Genetic Engineering and Biotechnology (ICGEB), Trieste, Italya; Rice Genomics Unit, Parco Tecnologico Padano, Localita Cascina Codazza, Lodi, Italyb

## Abstract

*Dickeya zeae* is an emerging rice (*Oryza sativa*) pathogen causing bacterial foot rot. Related pathogens affect maize (*Zea mays*) and potato (*Solanum tuberosum*) and a variety of important ornamental and floral plants. Here, we present the draft genome sequence of *D. zeae* DZ2Q, an isolate obtained from rice grown in Italy.

## GENOME ANNOUNCEMENT

A study conducted by the International Rice Research Institute (IRRI) showed that rice farmers lose 37% of rice yield due to pests (1). Increasing knowledge on rice pests is therefore important for finding solutions to reduce yield losses. An emerging pathogen is *Dickeya zeae* (formerly *Erwinia chrysanthemi* pv. zeae), the causal agent of bacterial foot rot in rice and of bacterial stalk rot in maize (2). *D. zeae* is a Gram-negative facultative anaerobic bacterium that is able to infect monocotyledons and dicotyledons. It causes soft rot in essential crops, such as *Zea mays*, *Oryza sativa*, *Solanum tuberosum*, and *Musa* spp., and in other economically important plants. *D. zeae* spreads via water, survives on plant debris and weeds, and colonizes the plant xylem, entering through injuries; high humidity and temperatures facilitate the development of the disease (3). *D. zeae* was first reported in Asia (4), and in recent years its incidence in rice fields has increased. In 2011, different rice-cultivated areas in Europe also reported foot rot infections (M. Biloni, personal communication).

We isolated *D. zeae* strain DZ2Q from diseased rice from a Roma cultivar grown in the Po Valley, and here, we present its draft genome sequence. Sequencing was performed using both Illumina (5) and 454-Roche (6) technologies. An Illumina GAII shotgun library (2,500,000 reads totaling 250.0 Mb) and a paired-end 454 GS-FLX library (407,289 reads totaling 152.3 Mb) were generated and sequenced. A total of 3,114,455 reads were obtained, with ~60-fold coverage of the 4.7-Mb genome. The *de novo* assembly was performed with MIRA version 3.4.0 (7) followed by manual curation, and this produced 26 contigs organized in 19 scaffolds. The genome of *D. zeae* DZ2Q has a G+C content of 53.4%, and according to automated annotation (8), it contains 4,649 predicted protein-coding sequences (CDSs). Of these, 85.1% were found in other species of *Dickeya*, while 4.5% of the genome was found to be strain specific compared with the two genomes of strains *D. zeae* ZJU1202 (9) and Ech1591 (http://genome.jgi-psf.org/dicda/dicda.info.html); in this portion of the *D. zeae* DZ2Q genome, a pathogenicity island and a complete prophage region were identified.

A first analysis of the DZ2Q genome revealed the presence of cell wall-degrading enzymes, and adjacent to the *luxI-luxR* type *N*-acylhomoserine lactone-based quorum-sensing (QS) system (10), the new QS system encoded by the *vfm* operon (11) was identified. Among the 72 CDSs identified by RAST as being involved in virulence, disease, and defense mechanisms (12), we identified the multidomain polyketide synthase gene for the production of zeamine, a phytotoxin and potent antibiotic (13) present also in the rice isolates *D. zeae* EC1 and *D. zeae* ZJU1202 but absent in maize isolate *D. zeae* Ech1591.

The genome sequence of this new isolate will provide genetic information for comparative analysis among different *D. zeae* strains, helping to understand *Dickeya* pathogenicity mechanisms and develop resistance strategies.

### Nucleotide sequence accession numbers.

This whole-genome shotgun project has been deposited at DDBJ/EMBL/GenBank under the accession no. APMV00000000. The version described in this paper is the first version, APMV01000000.
